# Outcomes of prophylactic abdominal aortic balloon occlusion in patients with placenta previa accreta: a propensity score matching analysis

**DOI:** 10.1186/s12884-022-04837-2

**Published:** 2022-06-20

**Authors:** Huifen Yin, Rong Hu

**Affiliations:** Obstetrics Department, Obstetrics and Gynecology Hospital of Fudan University, 419 Fangxie Rd, Shanghai, People’s Republic of China

**Keywords:** Placenta previa, Placenta accreta, Prophylactic abdominal aortic balloon occlusion, Propensity score analysis

## Abstract

**Background:**

Placenta previa accreta is a life-threatening pregnancy complication, and reducing blood loss during operative treatment remains a major challenge. The aim of our study was to investigate the effect of prophylactic abdominal aortic balloon occlusion (AABO) during caesarean section in women with placenta previa accreta.

**Methods:**

A retrospective study of women with placenta previa accreta was conducted in a tertiary hospital from January 1, 2015, to December 31, 2020. Women were divided into balloon and control groups by whether AABO was performed. Baseline characteristics and pregnancy outcomes were compared in the two groups. A propensity score analysis was applied to minimise the indication bias. The primary outcome was composite, including estimated blood loss (EBL) ≥ 2.0 L, massive transfusion and hysterectomy.

**Results:**

A total of 156 patients participated in this study, with 68 in the balloon group and 88 in the control group. Propensity score analysis showed that women in the balloon group had less EBL (1590.36 ± 1567.57 vs. 2830.36 ± 2285.58 mL, *P* = 0.02) as well as a lower proportion of EBL ≥ 1.0 L (50.00% vs. 78.57%, *P* = 0.03), EBL ≥ 2.0 L (21.43% vs. 50.00%, *P* = 0.03) and EBL ≥ 3.0 L (14.29% vs. 42.86%, *P* = 0.04). In addition, women in the control group received more red blood cell transfusions (8.43 U ± 9.96 vs. 3.43 U ± 6.27, *P* = 0.03), and the proportion of massive transfusions was higher (35.71% vs. 7.14%, *P* = 0.02). The proportions of disseminated intravascular coagulation (0% vs. 28.57%, *P* < 0.01), haemorrhagic shock (3.57% vs. 32.14%, *P* = 0.02) and hysterectomy (10.71% vs. 39.29%, *P* = 0.03) were significantly lower in the balloon group. Sutures were performed more often in the balloon group (64.29% vs. 17.86%, *P* < 0.01). Multivariate logistic regression analysis showed that AABO was associated with the primary outcome (adjusted odds ratio 0.46, 95% confidence interval 0.23 ~ 0.96, *P* = 0.04). No serious balloon catheter-related complications occurred in the balloon group.

**Conclusion:**

AABO was an effective and safe approach to improve maternal outcomes for patients with placenta previa accreta.

**Supplementary Information:**

The online version contains supplementary material available at 10.1186/s12884-022-04837-2.

## Introduction

The annual incidence of invasive placentation is estimated to be 1 in 300 pregnancies and has continuously increased over the last 50 years due to prior caesarean section, advanced maternal age and assisted reproductive technology [1]. Placenta accreta spectrum (PAS) is frequently associated with placenta previa [2], a life-threatening pregnancy complication. It may cause severe haemorrhaging, adjacent organ damage and infection, increasing maternal and neonatal mortality and morbidities [3]. Scheduled caesarean delivery near term is the management choice for women with placenta accreta in many clinical guidelines [4–6]. However, reducing blood loss and preserving fertility remain challenging during the operative treatment of placenta previa accreta.

Prophylactic abdominal aortic balloon occlusion (AABO) is a new technique to block blood perfusion in the uterus, reducing postpartum haemorrhage and the rate of hysterectomy [7–9]. However, the efficacy of AABO is uncertain because of the limited amount of literature and cases. Placement of balloon catheters may also cause complications such as thrombosis diseases, haematoma and rarely artery rupture [10–12]. Therefore, the objective of our study was to investigate whether AABO during caesarean section was effective and safe for women with placenta previa accreta.

## Methods

This retrospective study was conducted using medical records of women with placenta previa accreta in a single tertiary hospital from January 1, 2015, to December 31, 2020. Placenta previa was diagnosed through routine prenatal ultrasound in the third trimester by experienced ultrasonographers. Ultrasonographic diagnosis of placenta accreta was based on previously reported criteria [13–15] when at least one of the following abnormal ultrasound findings were present: loss of retroplacental clear zone, myometrial thinning, multiple placental lacunae, subplacental hypervascularity, turbulent flow in lacunae and bladder wall interruption. Magnetic resonance imaging (MRI) was performed according to the managing obstetrician’s discretion. Characteristics of MRI considered suggestive of placenta accreta included the following [16–18]: uterine bulging, intraplacental dark bands on T2-weighted imaging, heterogeneous placental signal, bladder tenting, placental protrusion into the cervix, or more than one of these characteristics. Placenta accreta was confirmed during surgery when there was a lack of spontaneous complete separation of the placenta from its basal plate, requiring manual removal or after direct visualization of placental tissue protruding through the uterine serosa. The degree of placenta accreta was diagnosed by clinical criteria and histologic criteria according to the International Federation of Gynecology and Obstetrics (FIGO) classification from 2018 [19]. The inclusion criteria were women diagnosed with placenta previa and suspicious accreta by ultrasound or MRI before caesarean delivery who were confirmed to have accreta after caesarean delivery. The exclusion criteria were multiple pregnancies and women with a delivery week < 28 weeks.

**Fig. 1:**
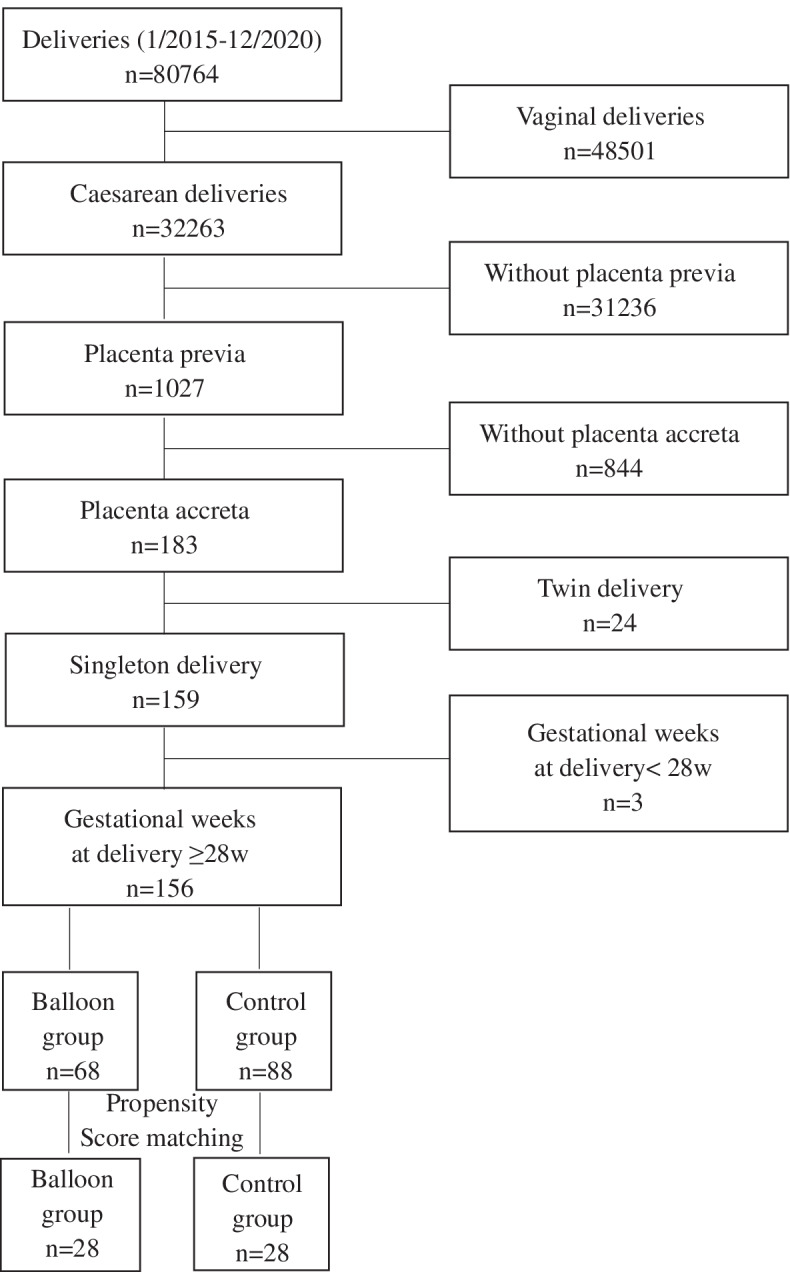
Patient flow chart

A total of 156 women participated in this study (Fig. 1). They were divided into a balloon group (*n* = 68) and a control group (*n* = 88) based on whether they received AABO. Prophylactic placement of balloon catheters was performed at the surgeon’s discretion. All women who received balloon catheters provided written informed consent. For all women in the control group, written informed consent was signed for caesarean delivery without prophylactic placement of balloon catheters. For women in the balloon group, the balloon catheter was performed before surgery, and the procedure was as follows. The catheter was inserted via the right femoral artery and placed at the abdominal aorta (above the division of the common iliac artery and under the renal ostia from the aorta) under fluoroscopic guidance with the balloon deflated. Then, the patient was transferred to the operating room for caesarean delivery. The balloon was inflated immediately after cord clamping. The balloon was inflated for no longer than 10 min and was then deflated for 1 min if the bleeding was active. If the bleeding became inactive, the balloon was kept deflated until the surgery was completed. After delivering the neonate, gentle controlled cord traction was performed in hopes of detaching the placenta if no placenta percreta was suspicious and no heavy bleeding happened. If removal of the placenta failed and the surgeons considered conservative management reasonable, surgical procedures including manual removal of the placenta, clamping and resecting the residual adherent tissue and the affected uterine wall, focal suturing on the placental detachment surface, and the uterine wall reconstruction were performed. Intraoperative haemostatic approaches such as uterine compression sutures, intrauterine gauze packing, intrauterine balloon tamponade and uterine artery ligation could be performed to control bleeding at the surgeon’s discretion. When all conservative therapies failed, the decision to perform a hysterectomy was made by at least two experienced surgeons. The catheter was removed after the surgery, and uterine artery embolisation was adopted if the patient was haemodynamically stable but exhibited persistent slow bleeding. For women in the control group, caesarean delivery was directly performed, and the perioperative management of the two groups was similar.

Maternal characteristics and pregnancy outcomes were collected. Maternal outcomes included operation time, estimated blood loss (EBL), autologous blood transfusion, homologous transfusion including packed red blood cells (PRBCs) transfusion, fresh frozen plasma (FFP) transfusion and other blood transfusions, intraoperative uterine compression suture, intrauterine gauze packing, intrauterine balloon tamponade, intraoperative bladder injury, hysterectomy, disseminated intravascular coagulation (DIC), haemorrhagic shock, uterine artery embolisation after surgery, relaparotomy, maternal length of antibiotic use, maternal length of hospital stay after surgery, complications of thromboembolism diseases, haematoma and artery rupture. The amount of intraoperative EBL was determined by adding blood volume in the surgical suction bottle (excluding amniotic fluid), that on gauze, and the visually estimated volume on the operating table. The primary outcomes included EBL ≥ 2.0 L, massive transfusion (transfusion of 10 or more units of packed red blood cells) and hysterectomy. Neonatal outcomes included gestational weeks at delivery, birthweight, Apgar score ≤ 7 at 1 min, Apgar score ≤ 7 at 5 min, neonatal intensive care unit (ICU) admission, assisted ventilation, pulmonary surfactant use, the complication of neonatal respiratory distress syndrome (NRDS), wet lung, neonatal asphyxia, neonatal infection, hyperbilirubinemia, antibiotic administration and neonatal length of hospital stay.

Data were analysed by SPSS statistical software version 22.0. Quantitative variables are presented as the mean ± standard deviation (SD), and qualitative variables are presented as frequencies and percentages. Continuous data were compared using Student’s t test or Wilcoxon rank-sum test for nonnormally distributed continuous variables. Categorical data were compared using the x^2^ test or Fisher’s exact test. A propensity score analysis with 1:1 matching was applied to minimise the indication bias of the two groups. Univariate analyses were adopted to screen characteristics with *P* < 0.2, and then a multivariable logistic regression model was adopted to acquire independent predictors for the composite primary outcome. *P* < 0.05 was considered statistically significant.

## Results

A total of 156 eligible women with placenta previa accreta participated in this study, and 68 (43.59%) received prophylactic abdominal aortic balloon before caesarean delivery. The characteristics of the entire cohort and propensity score-matched cohort are shown in Table 1. In the entire cohort, the proportions of gravidity ≥ 4 (36.76% vs. 57.95%, *P* = 0.01) and prior uterine curettage ≥ 3 (13.24% vs. 26.14%) were significantly different between the two groups. After propensity score matching, 28 women were included in each group, and all characteristics were comparable between the balloon and control groups.

**Table 1:** Baseline characteristics of two groups

	Entire cohort			Propensity score-matched cohort		
	Balloon(68)	Control(88)	*P* value	Balloon(28)	Control(28)	*P* value
Maternal age, y	33.38 ± 4.65	33.80 ± 4.13	0.56	33.79 ± 5.43	33.32 ± 4.41	0.73
BMI at delivery, kg/m^2^	26.54 ± 3.38	26.45 ± 3.75	0.87	26.25 ± 3.09	26.05 ± 2.50	0.80
Gravidity						
≤ 3	43(63.24%)	37(42.05%)	0.01*	13(46.43%)	15(53.57%)	0.59
≥ 4	25(36.76%)	51(57.95%)		15(53.57%)	13(46.43%)	
Parity						
≤ 1	59(86.76%)	75(85.23%)	0.78	22(78.57%)	24(85.71%)	0.73
≥ 2	9(13.24%)	13(14.77%)		6(21.43%)	4(14.29%)	
Prior uterine curettage						
≤ 2	59(86.76%)	65(73.86%)	0.048*	24(85.71%)	24(85.71%)	1.00
≥ 3	9(13.24%)	23(26.14%)		4(14.29%)	4(14.29%)	
Prior cesarean deliveries						
≤ 1	59(86.76%)	77(87.50%)	0.89	23(82.14%)	25(89.29%)	0.70
≥ 2	9(13.24%)	11(12.50%)		5(17.86%)	3(10.71%)	
History of placenta previa						
Yes	7(10.29%)	5(5.68%)	0.28	1(3.57%)	1(3.57%)	1.00
No	61(89.71%)	83(94.32%)		27(96.43%)	27(96.43%)	
History of postpartum hemorrhage						
Yes	6(8.82%)	1(1.14%)	0.06	1(3.57%)	1(3.57%)	1.00
No	62(91.18%)	87(98.86%)		27(96.43%)	27(96.43%)	
Hypertensive disorders						
Yes	3(4.41%)	6(6.82%)	0.77	2(7.14%)	2(7.14%)	1.00
No	65(95.59%)	82(93.18%)		26(92.86%)	26(92.86%)	
Gestational diabetes mellitus						
Yes	10(14.71%)	13(14.77%)	1.00	4(14.29%)	1(3.57%)	0.35
No	58(85.29%)	75(85.23%)		24(85.71%)	27(96.43%)	
Anterior placenta						
Yes	57(83.82%)	64(72.73%)	0.10	22(78.57%)	21(75.00%)	0.75
No	10(14.93%)	24(27.27%)		6(21.43%)	7(25.00%)	
Degree of PAS						
Grade 1 (Placenta accreta)	23(33.82%)	36(40.91%)	0.56	0(0.00%)	3(10.71%)	0.19
Grade 2 (Placenta increta)	38(55.88%)	46(52.27%)		16(57.14%)	13(46.43%)	
Grade 3 (Placenta percreta)	7(10.29%)	6(6.82%)		12(42.86%)	12(42.86%)	
Preoperative level of hemoglobin (g/dl)	110.28 ± 10.18	109.58 ± 11.50	0.69	110.39 ± 9.09	110.29 ± 12.38	0.97
Gestational age at delivery	35.98 ± 1.82	35.52 ± 2.49	0.21	35.98 ± 2.18	35.44 ± 2.70	0.41

*Abbreviations*: *BMI* Body mass index, *PAS* Placenta accreta spectrum

Values are given as mean ± SD or number (percentage)

^*^Significance values

Maternal outcomes are shown in Table 2. In the propensity score-matched cohort, women in the balloon group had significantly less EBL (1590.36 ± 1567.57 vs. 2830.36 ± 2285.58 mL, *P* = 0.02) and a lower proportion of EBL ≥ 1.0 L (50.00% vs. 78.57%, *P* = 0.03), EBL ≥ 2.0 L (21.43% vs. 50.00%, *P* = 0.03) and EBL ≥ 3.0 L (14.29% vs. 42.86%, *P* = 0.04). The number of RBC transfusions in the control group was significantly higher (8.43 ± 9.96 vs. 3.43 ± 6.27, *P* = 0.03), and the proportion of massive transfusions was also higher (35.71% vs. 7.14, *P* = 0.02). The proportions of DIC (0% vs. 28.57%, *P* < 0.01), haemorrhagic shock (3.57% vs. 32.14%, *P* = 0.02) and hysterectomy (10.71% vs. 39.29%, *P* = 0.03) were significantly lower in the balloon group. Sutures were performed more often in women with balloon placement during caesarean Sect. (64.29% vs. 17.86%, *P* < 0.01). No serious balloon catheter-related complications, including thromboembolic disease, haematoma or artery rupture, occurred in women with balloon occlusion. Neonatal outcomes are shown in Table 3, and there were no significant differences between the two groups in the propensity score-matched cohort (*P* > 0.05).

**Table 2:** Maternal outcomes of women in the two groups

Variable	Balloon(68)	Control(88)	*P* value	Balloon(28)	Control(28)	*P* value
Operation time, min	98.66 ± 43.52	100.56 ± 54.72	0.82	100.18 ± 48.07	111.00 ± 57.29	0.45
EBL, L	1604.56 ± 1481.37	2078.32 ± 1822.87	0.08	1590.36 ± 1567.57	2830.36 ± 2285.58	0.02*
EBL ≥ 1.0L	36 (52.94%)	54 (61.36%)	0.29	14(50.00%)	22(78.57%)	0.03*
EBL ≥ 2.0L	16 (23.53%)	35 (39.77%)	0.03*	6(21.43%)	14(50.00%)	0.03*
EBL ≥ 3.0L	11 (16.18%)	26 (29.55%)	0.05	4(14.29%)	12(42.86%)	0.04*
Transfusion						
Proportion of homologous transfusion	48 (70.59%)	62 (70.45%)	0.99	17(60.71%)	24(85.71%)	0.07
Autologous blood	402.81 ± 442.72	279.68 ± 452.46	0.09	369.57 ± 429.85	362.11 ± 501.39	0.95
RBC, U	3.96 ± 6.00	5.55 ± 7.13	0.14	3.43 ± 6.27	8.43 ± 9.96	0.03*
FFP, mL	492.71 ± 632.25	567.05 ± 660.32	0.48	435.86 ± 606.17	825.00 ± 915.56	0.07
Cryoprecipitate, U	7.09 ± 9.66	6.53 ± 8.31	0.70	7.21 ± 11.46	8.21 ± 9.18	0.72
Total transfusion	1783.82 ± 2125.00	1912.13 ± 2134.35	0.71	1607.21 ± 2212.48	2730.32 ± 2777.92	0.72
Massive transfusion	8(11.76%)	22(25.00%)	0.04*	2(7.14%)	10(35.71%)	0.02*
DIC	3(4.41%)	15(17.04%)	0.03*	0(0.00%)	8(28.57%)	< 0.01*
Hemorrhagic shock	6(8.82%)	16(18.39%)	0.09	1(3.57%)	9(32.14%)	0.02*
Hysterectomy	12(17.65%)	23(26.14%)	0.21	3(10.71%)	11(39.29%)	0.03*
Bladder injury	2(2.94%)	3(3.41%)	0.87	0(0.00%)	1(3.57%)	1.00
Intrauterine gauze packing/balloon tamponade	43((63.24%)	52(59.09%)	0.60	15(53.57%)	19(67.86%)	0.27
Intraoperative uterine compression suture	35(51.47%)	27(30.68%)	0.01*	18(64.29%)	5(17.86%)	< 0.01*
Uterine artery embolization after surgery	30(44.12%)	20(22.73%)	< 0.01*	8(28.57%)	7(25.00%)	0.76
Relaparotomy	5(7.35%)	8(9.09%)	0.68	1(3.57%)	5(17.86%)	0.20
Length of maternal antibiotic use, d	4.71 ± 3.16	3.97 ± 2.43	0.11	4.25 ± 2.66	4.50 ± 2.86	0.74
Length of maternal stay after surgery, d	7.66 ± 4.42	6.51 ± 2.82	0.05	7.29 ± 4.22	6.68 ± 3.00	0.54
Thromboembolism diseases	0	0	NA	0	0	NA
Hematoma	0	0	NA	0	0	NA
Artery rupture	0	0	NA	0	0	NA

*Abbreviations*: *EBL* Estimated blood loss, *RBC* Red blood cells, *FFP* Fresh frozen plasma, *DIC* Disseminated intravascular coagulation

Values are given as mean ± SD or number (percentage)

^*^Significance values

**Table 3:** Neonatal outcomes of women in the two groups

Variable	Balloon(68)	Control(88)	*P* value	Balloon(28)	Control(28)	*P* value
Gestational weeks at delivery, wk	35.98 ± 1.82	35.52 ± 2.49	0.21	35.98 ± 2.18	35.44 ± 2.70	0.41
Birthweight, g	2778.75 ± 543.86	2617.40 ± 594.53	0.08	2749.82 ± 591.75	2571.61 ± 613.86	0.27
Apgar score ≤ 7 at 1 min	19(27.94%)	6(6.82%)	< 0.01*	6(21.43%)	2(7.14%)	0.25
Apgar score ≤ 7 at 5 min	2(2.94%)	2(2.27%)	1.00	2(7.14%)	1(3.57%)	1.00
Neonatal ICU admission	45(66.18%)	46(52.27%)	0.08	18(64.29%)	14(50.00%)	0.28
Assisted ventilation	38(55.88%)	38(43.18%)	0.12	15(53.57%)	12(42.86%)	0.42
Pulmonary surfactant use	13(19.12%)	25(28.41%)	0.18	4(14.29%)	7(25.00%)	0.31
NRDS	12(17.65%)	24(27.27%)	0.16	4(14.29%)	6(21.43%)	0.73
Wet lung	26(38.24%)	13(14.77%)	< 0.01*	11(39.29%)	5(17.86%)	0.08
Neonatal asphyxia	19(27.94%)	6(6.82%)	< 0.01*	7(25.00%)	2(7.14%)	0.15
Neonatal infection	5(7.35%)	13(14.77%)	0.15	3(10.71%)	4(14.29%)	1.00
Antibiotic administration	17(25.00%)	26(29.55%)	0.53	6(21.43%)	8(28.57%)	0.54
Hyperbilirubinemia	32(47.06%)	28(31.82%)	0.05	12(42.86%)	7(25.00%)	0.16
Length of neonatal hospital stay, d	8.67 ± 7.43	9.24 ± 8.85	0.74	8.22 ± 8.61	10.86 ± 10.65	0.45

*Abbreviations*: *ICU* Intensive care unit, *NRDS* Respiratory distress syndrome

Values are given as mean ± SD or percentage

^*^Signigicance values

Univariate analysis showed that the degree of PAS and AABO were potentially associated with the primary outcome (*P* < 0.02) (Table 4). Multivariate logistic regression analysis showed that AABO was associated with the primary outcome (adjusted odds ratio 0.46, 95% confidence interval 0.23 ~ 0.96, *P* = 0.04) (Table 4).

**Table 4:** Univariate and multivariate logistic analysis of the entire cohort for the primary outcome

Variable	Univariate analysis		Multivariate logistic analysis	
	OR (95% CI)	*P* value	OR (95% CI)	*P* value
Maternal age, y	1.02(0.94 ~ 1.10)	0.69	NA	NA
BMI at delivery, kg/m^2^	1.06(0.96 ~ 1.16)	0.24	NA	NA
Gravidity				
≤ 3	1	Reference	NA	NA
≥ 4	1.14(0.59 ~ 2.22)	0.69	NA	NA
Parity				
≤ 1	1	Reference	NA	NA
≥ 2	1.13(0.44 ~ 2.89)	0.80	NA	NA
Prior uterine curettage				
≤ 2	1	Reference	NA	NA
≥ 3	1.22(0.54 ~ 2.72)	0.64	NA	NA
Prior cesarean delivery				
≤ 1	1	Reference	NA	NA
≥ 2	1.35(0.52 ~ 3.53)	0.54	NA	NA
History of placenta previa	2.06(0.63 ~ 6.74)	0.22	NA	NA
History of postpartum hemorrhage	1.49(0.32 ~ 6.89)	0.92	NA	NA
Hypertensive disorders	0.97(0.23 ~ 4.04)	1.00	NA	NA
Diabetes	0.83(0.32 ~ 2.16)	0.70	NA	NA
Anterior placenta	1.16(0.52 ~ 2.60)	0.72	NA	NA
Degree of PAS			2.73(1.50 ~ 4.96)	< 0.01*
Grade 1 (Placenta accreta)	1	Reference		
Grade 2 (Placenta increta)	2.53(1.17 ~ 5.48)	0.02		
Grade 3 (Placenta percreta)	6.27(1.73 ~ 22.64)	< 0.01*		
Preoperative level of hemoglobin	0.98(0.95 ~ 1.01)	0.27	NA	NA
Intra-aortic balloon occlusion	0.55(0.27 ~ 1.08)	0.08	0.46(0.23 ~ 0.96)	0.04

*Abbreviations*: *BMI* Body mass index, *PAS* Placenta accreta spectrum

Primary outcome includes estimated blood loss ≥ 2.0L, massive transfusion and hysterectomy

^*^Significance values

## Discussion

In our study, propensity score analysis and multivariate logistic regression analysis were used to investigate over 150 cases of placenta previa and accreta. The results showed that prophylactic AABO effectively reduced blood loss and blood transfusion, decreasing the risk of DIC, haemorrhagic shock and hysterectomy; and did not influence neonatal outcomes. No related complications occurred in any of the patients. However, intraoperative uterine compression sutures were more commonly used in women in the balloon group. Multivariate logistic regression analysis also showed that AABO was associated with women’s primary outcomes.

Management by a multidisciplinary team for women with placenta previa accreta is imperative because it may cause massive bleeding and be life-threatening [20]. Prenatal diagnosis of PAS is usually accomplished by ultrasound whereas prenatal MRI is commonly performed to diagnosis and describe the depth and topography of placental invasion [2]. Then a pre-planned treatment of PAS could be given to improve maternal outcomes. In recent decades, some intravascular interventional therapies, including bilateral internal iliac artery balloon occlusion (IIABO) and AABO, have been used to reduce intraoperative bleeding and decrease the rate of hysterectomy. However, some studies found that the use of IIABO did not improve maternal outcomes in women with placenta previa accreta [21–23], and one study suggested that blocking blood flow in the internal iliac arteries was not enough because blood flow from the external iliac arteries was also observed in placenta previa accreta [24]. On the other hand, the efficacy of AABO is uncertain because of the limited amount of literature and cases. Chen et al. suggested that ABBO was effective in reducing postpartum haemorrhage and blood transfusion and decreasing the risk of hysterectomy, but intrauterine gauze packing was more commonly used in the balloon group [25]. Wang et al. found that ABBO was an effective way to control intraoperative blood loss and blood transfusion and decrease the risk of haemorrhagic shock. However, the rate of hysterectomy was not decreased [26]. Our study showed that AABO effectively reduced blood loss and blood transfusion and decreased the risk of DIC, haemorrhagic shock and hysterectomy, which was partially in accordance with previous studies. Our study also showed that intraoperative uterine compression sutures were more commonly used in women in the balloon group. Solely from a surgical viewpoint, the drier the surgical field is, the better it is. Profuse and brisk bleeding commonly occurs during manual removal of the placenta. It can be prevented by applying AABO, thus making it possible for the operator to complete the oversewing and curettage of the placenta implantation site with acceptable blood loss [27] and eventually decreasing the risk of DIC haemorrhagic shock and hysterectomy.

On the other hand, AABO is an invasive technique. It may cause complications, including initial vessel injury, arterial thrombosis, puncture point haematoma, ischaemic necrosis of the lower limbs, reperfusion injury of tissues and organs, acute renal failure and rarely artery rupture [10–12, 28]. It was reported that measurement of the abdominal aortic diameter before the intervention, clearance of the opening of the renal artery and control of the duration of balloon occlusion are essential for decreasing the risk of complications [29]. In our study, the diameter of the abdominal aorta was measured by MRI before surgery to determine the appropriate size of the balloon catheter. Occlusion was maintained for 10 min at a time with an interval of 1 min during surgery. Eventually, no complications occurred in any of the patients. In addition, for short-term neonatal outcomes, the results showed no difference between the two groups. Additionally, minimising foetal radiation exposure was also a favourable aspect for this method. In our study, balloon insertion was performed rapidly by an experienced radiologist. The foetal radiation exposure dose was less than 20 mGy, far less than the standard dose of ≤ 150 mGy recommended by the National Committee on Radiological Protection [30].

Our study had some strengths. First, the number of cases was much larger than that in previous studies (usually no more than 100) to perform propensity score analysis to minimise the indication bias for the decision to use AABO based on the surgeon’s discretion. In addition, the maternal and neonatal outcomes compared in our study were comprehensive. There were also some limitations of our study. This study was retrospectively designed in a single centre and lacked long-term follow-up. In the future, multicentre prospective investigations should be performed with more extended follow-up periods to provide accurate assessment and validation of the clinical efficacy of this method.

## Conclusion

AABO was an effective and safe approach to improve maternal outcomes for patients with placenta previa accreta without increasing complications, and it does not cause harm to the newborn.

## Supplementary Information

**Additional file 1.**

## Data Availability

All data generated or analysed during this study are included in this published article and its supplementary information files.

## Abbreviations

PAS: Placenta accreta spectrum
AABO: Abdominal aortic balloon occlusion
EBL: Estimated blood loss
MRI: Magnetic resonance imaging
FIGO: International Federation of Gynecology and Obstetrics
PRBCs: Packed red blood cells
FFP: Fresh frozen plasma
DIC: Disseminated intravascular coagulation
NRDS: Neonatal respiratory distress syndrome
SD: Standard deviation
IIABO: Iliac artery balloon occlusion
BMI: Body mass index
ICU: Intensive care unit
